# Hematopoietic-Prostaglandin D2 synthase through PGD2 production is involved in the adult ovarian physiology

**DOI:** 10.1186/1757-2215-4-3

**Published:** 2011-02-25

**Authors:** Andalib Farhat, Pascal Philibert, Charles Sultan, Francis Poulat, Brigitte Boizet-Bonhoure

**Affiliations:** 1 Institut de Génétique Humaine, Department of Genetic and Development, CNRS UPR1142, 141, rue de la Cardonille, 34396 Montpellier CEDEX5, France; 2 Service d'Hormonologie, Hôpital Lapeyronie, CHU Montpellier, France

## Abstract

**Background:**

The prostaglandin D2 (PGD2) pathway is involved in numerous biological processes and while it has been identified as a partner of the embryonic sex determining male cascade, the roles it plays in ovarian function remain largely unknown. PGD2 is secreted by two prostaglandin D synthases (Pgds); the male-specific lipocalin (L)-Pgds and the hematopoietic (H)-Pgds.

**Methods:**

To study the expression of the Pgds in the adult ovary, *in situ *hybridization were performed. Then, to evaluate the role of H-Pgds produced PGD2 in the ovarian physiology, adult female mice were treated with HQL-79, a specific inhibitor of H-Pgds enzymatic activity. The effects on expression of the gonadotrophin receptors *FshR *and *LhR*, steroidogenic genes *Cyp11A1*, *StAR *and on circulating progesterone and estradiol, were observed.

**Results:**

We report the localization of *H-Pgds *mRNA in the granulosa cells from the primary to pre-ovulatory follicles. We provide evidence of the role of H-Pgds-produced PGD2 signaling in the FSH signaling through increased *FshR *and *LhR *receptor expression. This leads to the activation of steroidogenic *Cyp11A1 *and *StAR *gene expression leading to progesterone secretion, independently on other prostanoid-synthetizing mechanisms. We also identify a role whereby H-Pgds-produced PGD2 is involved in the regulation of follicular growth through inhibition of granulosa cell proliferation in the growing follicles.

**Conclusions:**

Together, these results show PGD2 signaling to interfere with FSH action within granulosa cells, thus identifying an important and unappreciated role for PGD2 signaling in modulating the balance of proliferation, differentiation and steroidogenic activity of granulosa cells.

## Background

Folliculogenesis is under the control of growth factors and two pituitary gonadotropin hormones; follicle-stimulating hormone (FSH) and luteinizing hormone (LH). These heterodimeric glycoproteins bind in the ovary to specific G-protein coupled receptors, FshR and LhR respectively, to facilitate the growth and differentiation of ovarian cells and also to control the production of the two steroid hormones estradiol and progesterone, for review see [1,2].

Amongst the several autocrine and/or paracrine growth factors produced by the follicle itself, prostaglandins are critical for multiple stages of reproduction [3,4]. Mice lacking the *cyclo-oxygenase-2 *(*Cox-2*) gene encoding the rate limiting step in prostaglandin synthesis, show pre-implantation deficiencies throughout ovulation and fertilization [5]. This phenotype is also seen in the absence of prostaglandin E2 (PGE2) receptor EP2 [6]. A surge in LH levels in granulosa cells of pre-ovulatory follicles induces expression of *Cox-2 *and EP2 [7], while elevated PGE2 in turn, stimulates cumulus expansion by elevating cAMP [8]. It has also been shown that PGE2 increases expression of the *aromatase Cyp19A1 *gene, the key gene in estrogen biosynthesis in granulosa cells [9], as well as acting as a luteotrophic component to stimulate luteal progesterone secretion through a cAMP-mediated pathway in both human and ruminants [10]. Besides PGE2, prostaglandin PGF2α secretion via *cyclo-oxygenase COX-1 *expression and the action of its receptor FP, also plays an important role in diminishing progesterone levels and stimulating luteolysis, a crucial stage in inducing labor and pup delivery during parturition in human and mice [11,12]. Whereas PGE2 and PGF2α are both involved in regulating ovulation, luteinization, luteolysis and fertility [13-16], the role(s) of PGD2 signaling in folliculogenesis and ovarian physiology is not precisely understood.

PGD2 has been implicated as a signaling molecule in the mediation or regulation of various biological processes such as platelet aggregation, broncho-constriction and allergic diseases [17,18], whilst also being identified as a partner of the embryonic sex-determining male cascade [19,20]. Secreted PGD2 interacts with two receptors: (i) the specific membrane-bound DP receptor (DP1) associated with adenylcyclase and intracellular cAMP production [21,22], and (ii) chemo attractant receptor Th2 (CRTH2) cells (DP2) which is coupled to Ca^2+ ^signaling. A metabolite of PGD2, PGJ2, has also been shown to bind the peroxisome proliferator-activated receptor PPARγ a member of the orphan nuclear receptor superfamily implicated in key female reproductory roles [23]. PGD2 is produced by two prostaglandin D synthases (Pgds) responsible for mediating the final regulatory step in the biosynthetic pathway of PGD2 production [24]: (i) the lipocalin-type Pgds (L-Pgds), a member of the lipocalin ligand-carrier protein family [24,25] and (ii) the hematopoietic-type Pgds (H-Pgds) or GSH-requiring enzyme [26].

The *L-Pgds *transcript initially found in the brain [27], represents one of the ten most abundant transcripts in the cortex, hypothalamus and pituitary gland [28]. However, it is not expressed in either the embryonic or the adult ovary [20,29,30] whereas *H-Pgds *is expressed in the embryonic gonad of both sexes (submitted data). H-Pgds is a cytosolic protein responsible for the biosynthesis of PGD2 in immune and inflammatory cells such as mast cells or Th2 cells, and is also expressed in the spleen, thymus, skin and liver [26], in the microglia where H-Pgds-produced PGD2 is responsible for the neuroinflammation associated with brain injury and neurodegenerative diseases [31], as well as in trophoblasts, uterine epithelium and endometrial glands at the implantation site of the human decidua [32]. H-Pgds expression was also found in the hypothalamus-pituitary axis of hens and has been associated with high egg production [33]. Recently, PGD2 produced by H-Pgds and its metabolite PGJ2 have been shown to induce transcription of the *Lhb *subunit gene in the primary culture of chicken anterior pituitary cells, via the PPARα and PPARγ signaling pathways [34]. On the other hand, a stimulatory effect of PGD2 on progesterone secretion has been found *in vitro *in isolated human corpus lutea [35]. However, the precise *H-Pgds *expression profile and function of PGD2 signaling in the adult ovary remain unknown.

Here, we report the characterization and ovarian localization of *H-Pgds *mRNA and provide evidence of a role of H-Pgds-produced PGD2 signaling in the FSH signaling via the increase of *FshR *and *LhR *receptor expression, leading to activation of steroidogenic *Cyp11A1 *and *StAR *gene expression and progesterone secretion. We found that *in vivo *inhibition of H-Pgds activity failed to modify PGE2 and PGF2α synthesis in the ovary and also identify a role for H-Pgds-produced PGD2 in follicular growth regulation. Our results provide evidence that PGD2 signaling is a modulator of the differentiation and steroidogenic activity of granulosa cells.

## Methods

### Mouse strain and treatments

Female C57BL/6J mice (Charles River Laboratories, Saint Germain sur l'Arbresle, France) were housed at the IGH animal care facility under controlled environmental conditions (12 h light/12 h darkness, temperature 21°C). Animal care and handling conformed to the Réseau des Animaleries de Montpellier (RAM) and all procedures were approved by the Languedoc-Roussillon Regional Ethic committee (permit number 34-366, 2008 to BBB).

HQL-79 (4-benzhydryloxy-1-[3-(1*H*-tetrazol-5-yl)-propylpiperidine) [36], an inhibitor of H-Pgds activity, was purchased from Cayman Chemical (SpiBio, Interchim Montluçon, France). A HQL-79 solution (2.5 mg/ml) was made in methanol as recommended by the supplier and diluted to 0.125 mg/ml in 0.6% saline solution. Daily oral administration of HQL-79 was performed on 8 weeks old- cycling female mice for 5 to 9 days (for ovaries analyzis at the estrous phase) or for 16 days (for study of the length of the estrous cycle (three to four cycles)), as mentioned in the text. According to previous studies [36-38], 0.1, 1 or 10 mg/kg/day were administrated for the first experiment and then 1 mg/kg/day was administrated in the following experiments since the three doses had the same impact on the expression of ovarian markers. As a control, the same volume of vehicle (0.5% methanol) was orally administrated into control cycling mice during the same period.

Young cycling female mice (6 weeks) were treated with 5 I. U. PMSG (pregnant mare serum gonadotropin, Sigma-Aldrich, St Louis, MO, USA) without or with administration of HQL-79 inhibitor (1 mg/kg/day). PMSG was dissolved in 0.6% saline solution and injected s.c. in a total volume of 0.1 ml, at the diestrous or proestrous stages of the cycle to initiate follicular development. Ovaries were dissected 48 h later for analysis.

### Determination of estrous cycle

To determine the stages of estrous cycle, vaginal washes were collected for 16 days (three to four cycles) from five wild type (WT) and five HQL-79 mice. Diestrous phase was defined by the exclusive presence of leukocytes; proestrous phase by leukocytes and nucleated epithelial cells; estrous phase by large and squamous-type epithelial cells without nuclei; and metestrous by leukocytes and epithelial cells with translucent nuclei.

### Histology, immunofluorescence and in situ hybridization

For each female mouse, one ovary was processed for immunofluorescence and the other one was subjected to quantitative RT-PCR. Tissues were fixed in 4% paraformaldehyde at 4°C overnight and then embedded in OCT [39]. Cryosections (10 mm) were processed for immunofluorescence, after rehydration. Sections were then incubated overnight at room temperature with primary antibodies at the indicated dilutions: rabbit anti-CYP11A1 (1/200 dilution, gift of Dr Nadia Cherradi, CEA Grenoble) [40], rabbit anti-phospho-histone H3 (1/100 dilution, sc-8656, Santa Cruz Biotechnology, SantaCruz, CA, USA)), rat anti-H-Pgds (1/100 dilution, Cayman Chemical (SpiBio, France)), mouse anti- laminin (1/500 dilution, Sigma Aldrich), goat anti-FOXL2 (1/100 dilution, Santa Cruz Biotechnology) and goat anti-AMH (1/200 dilution, sc- 6886, Santa Cruz Biotechnology). After washing, sections were incubated with appropriate secondary antibodies (1/800 dilution, Alexa) (Molecular Probes, Invitrogen, Carlsbad, CA, USA) for 40 min.

The antisense *H*-*Pgds *and *FoxL2 *RNA probes were PCR-amplified from embryonic mouse cDNAs, cloned in a pCRII Topo vector (Invitrogen) and sequenced using an ABI automatic sequencer. Digoxigenin-labeled riboprobes were synthesized using a digoxigenin RNA labeling kit, following the manufacturer's instructions (Roche Diagnostics, Indianapolis, IN, USA) and used for *in situ *hybridization experiments on cryosections of WT ovaries, as previously described [20,41].

### RNA isolation and quantitative RT-PCR analysis of gene expression

RNA isolation using the RNeasy Midi kit (Qiagen, Valencia, CA, USA) from frozen ovaries, reverse transcriptase and quantitative RT-PCR using a LightCycler480 apparatus (Roche Diagnostics) were carried out as previously described [20]. Gene expression levels were investigated using different pairs of primers (Table 1) and normalized to those of *Gapdh *or *Hprt *.

**Table 1:** Sequences of oligonucleotides for real time PCR

Primers	Sequence 5'-3'	Primers	Sequence 5'-3'
mFSHRfwd	gtgcgggctactgctacact	mGapdhFwd	tggcaaagtggagattgttgcc
mFSHRrev	caggcaatcttacggtctcg	mGapdhRev	aagatggtgatgggcttcccg
mLHRqFwd	gatgcacagtggcaccttc	mP27Fwd	gagcagtgtccagggatgag
mLHRqRev	cctgcaatttggtggaagag	mP27Rev	tctgttctgttggccctttt
mStARqFwd	ttgggcatactcaacaacca	mCycD2Fwd	ctgtgcatttacaccgacaac
mStARqRev	acttcgtccccgttctcc	mCycD2Rev	cactaccagttcccactccag
mSCCqFwd	aagtatggccccatttacagg	mCox-1Fwd	cctctttccaggagctcaca
mSCCqRev	tggggtccacgatgtaaact	mCox-1Rev	tcgatgtcaccgtacagctc
mDP1Fwd	cccagtcaggctcagactaca	mCox-2Fwd	gctcttccgagctgtgct
mDP1Rev	aagtttaaaggctccatagtacgc	mCox-2Rev	cggttttgacatggattgg
mDP2Fwd	catcgtggttgccttcgt	mPges-2Fwd	cccaggaaggagacagctt
mDP2Rev	gcctccagcagactgaagat	mPges-2Rev	aggtaggtcttgagggcactaat
mSF-1Fwd	cacgaaggtgcatggtctt	mHPgdsFwd	cacgctggatgacttcatgt
mSF-1Rev	cagttctgcagcagtgtcatc	mHpgdsRev	aattcattgaacatccgctctt
mCYP19Fwd	cctcgggctacgtggatg	mLPgdsFwd	ggctcctggacactacacct
mCYP19Rev	gagagcttgccaggcgttaaa	mLPgdsRev	atagttggcctccaccactg
mEP2Fwd	tgctccttgcctttcacaat	mFPFwd	ctggccataatgtgcgtct
mEP2Rev	ctcggaggtcccacttttc	mFPRev	tgcaatgttggccattgtta
hGapdhFwd	gagaaggctggggctcat	hHPgdsFwd	gagaatggcttattggtaactctgt
hGapdhRev	tgctgatgatcttgaggctg	hHPgdsRev	aaagaccaaaagtgtggtactgc

### Hormone and prostaglandin assays

Hormone assays for estradiol and progesterone were performed from sera, by using ELISA kits (Cayman Chemicals, Progesterone EIA kit 582601 and Estradiol EIA kit 582251). Mice (n = 20 for WT and n = 20 for HQL-79-treated) at the estrous phase of their cycle, were anesthetized and blood was collected by cardiac puncture into plastic eppendorf tubes containing heparin. After centrifugation, the serum was extracted twice with methylene chloride; after evaporation, steroid extracts were stored at -80°C until assays were performed. Determination of the hormone concentrations was performed in triplicate at two different dilutions according to the kits'manufacturer. In each case, the twenty values were averaged.

PGD2, PGE2 and PGF2α levels were determined using the PGD2 - MOX EIA Kit (Cayman Chemical 500151), PGE2 express EIA kit (500141, Cayman Chemical) and 13,14-dihydro-15keto PGF2α (516671, Cayman Chemical), respectively. Ovaries were collected from mice treated (n = 8) or not (n = 8) by HQL-79 and immediately frozen on dry ice and then stored at -80°C. Ovaries were lyzed and proteins were extracted with cold acetone on ice and lyzates were evaporated under nitrogen flow. Prostaglandins were resuspended in 500 μl EIA buffer and assayed as recommended by the kits supplier. Two dilutions (1 and 1/20) were assayed for prostaglandins content. The eight values for each group were averaged and statistical analysis was performed using Student's *t *test, and results were considered statistically significant at a *P *< 0.05.

### Statistical analysis

Quantified real time RT-PCR signals were normalized to *Gapdh *or *Hprt *levels and the hormone levels of treated ovaries were compared to those of untreated ovaries. All values were presented as means ± SE. Student's t test was used to determine the significance of differences in expression and hormone data. Results were considered significant at P < 0.05 for two-sided analysis.

## Results

### *H-Pgds *and DP2 expression in adult mouse ovaries

**Figure 1:**
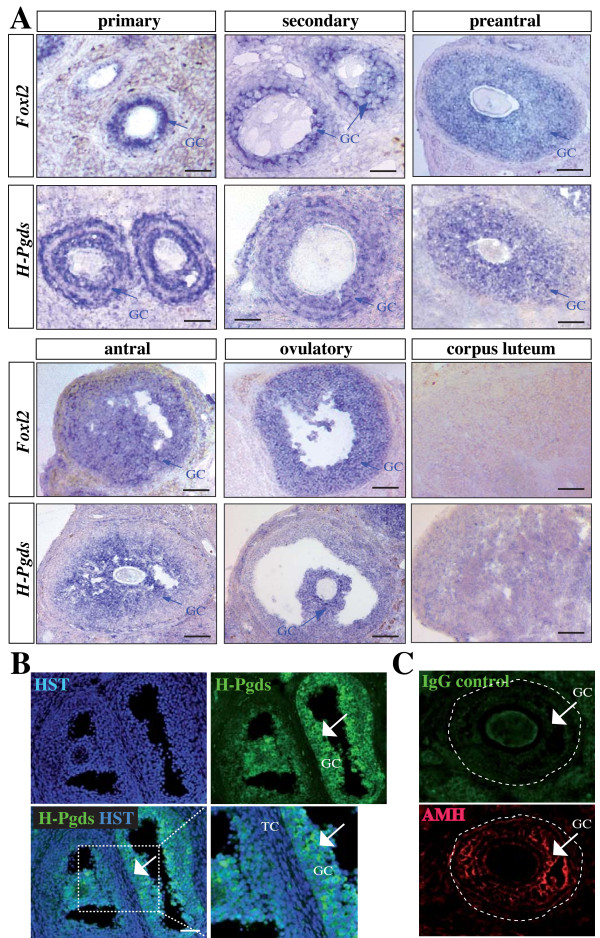
**Expression of H-*Pgds *in the mouse adult ovary**. (**A**), *In situ *hybridization for *H-Pgds *and granulosa cell marker *FoxL2 *was performed on sections from wild type adult ovaries. Primary, secondary, pre-antral, antral, ovulatory follicles and corpus luteum are represented for *H-Pgds *and *FoxL2 *mRNA expression and expressing granulosa cells (GC) are labeled by a blue arrow. Scale bars = 50 μm. (**B**), H-Pgds protein expression was detected in granulosa cells on wild type adult ovary sections, using an anti-H-Pgds antibody (in green) whereas nuclei are labeled in blue by the Hoescht Dye (HST). The merge panel has been enlarged on the right bottom panel. Arrows indicate H-Pgds expressing granulosa cells. TC, theca cells; GC, granulosa cells. Scale bar = 50 μm. (**C**), Control immunofluorescence experiment with no primary H-Pgds antibody (IgG control) showing the specificity of the antibody. AMH staining in granulosa cells was used on the same slide. Arrows indicate granulosa cells (GC).

**Figure 2:**
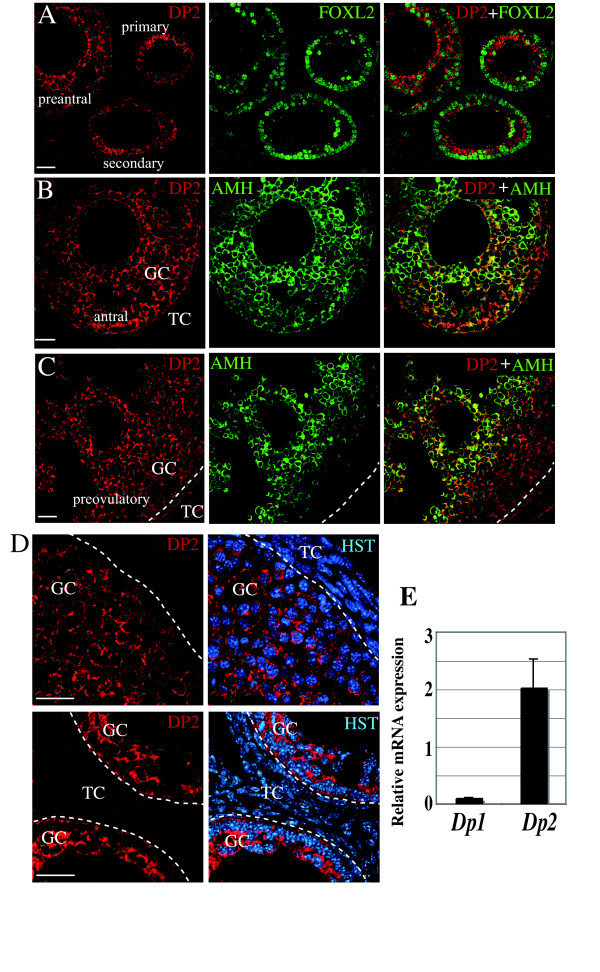
**Expression of PGD2-receptors in the mouse adult ovary**. DP2 protein expression was detected in granulosa cells of primary, secondary and preantral follicles (**A**), of antral **(B) **and preovulatory **(C) **follicles of wild type adult ovary, using immunofluorescence staining with an anti-DP2 antibody (in red) whereas FOXL2 **(A) **or AMH **(B,C) **(in green) were used to delineate granulosa cells. Right panels are the merge between DP2 and FOXL2 or AMH stainings. Dotted lines delineate granulosa (GC) and theca (TC) cells. Scale bars = 50 μm. (**D**), Control immunofluorescence experiment using an anti-DP2 antibody with the Hoescht dye (HST) labeling nuclei. Dotted lines delineate granulosa (GC) and theca (TC) cells within a follicle. Scale bars = 25 μm. (**E**), Expression levels of PGD2 receptors *Dp1 *and *Dp2 *mRNAs by real time RT-PCR. *Dp2 *was expressed at high levels in ovaries from adult cycling mice (n = 4) whereas *Dp1 *transcripts were undetectable. The values of three repeats were averaged and normalized to *Gapdh *expression.

The mRNA for *H-Pgds *was detected by *in situ *hybridization in the growing follicles from the primary to the pre-ovulatory stage and in the corpus luteum. Figure 1A shows an expression of *H-Pgds *mRNA in the granulosa cells of the developing follicles similar to that of the granulosa cell marker *FoxL2 *whereas hybridization with the control sense *H-Pgds *cRNA probe showed no significant signal (data not shown). In the antral and pre-ovulatory follicles, *H-Pgds *expression is likely abolished in the external layers of mural granulosa cells, remaining only in the internal layers of granulosa cells and in granulosa cells forming the cumulus in the ovulatory follicle. *H-Pgds *mRNA was not detectable in the other ovarian cell types. In order to confirm H-Pgds expression in the granulosa cells at the protein level, we used immunofluorescence with (Figure 1B, arrows) or without (Figure 1C, IgG control) a specific H-Pgds antibody. We then showed the DP2 receptor expression in the granulosa cells of primary, secondary, preantral (Figure 2A), antral (Figure 2B) and preovulatory (Figure 2C) follicles using an anti-rabbit DP2 antibody together with anti-FOXL2 (A) or anti-AMH (B, C) antibodies, two specific granulosa markers. Specific expression of DP2 in the granulosa cells was confirmed by high magnification imaging (Figure 2D). However, DP1 receptor was not detected in any cell type at any stage (data not shown). Indeed, using real-time RT-PCR we observed significant levels of *Dp2 *transcripts (Figure 2E), whereas *Dp1 *expression level remained undetectable in WT ovaries.

### Prostaglandin synthesis in the ovary upon inhibition of H-Pgds enzymatic activity

**Figure 3:**
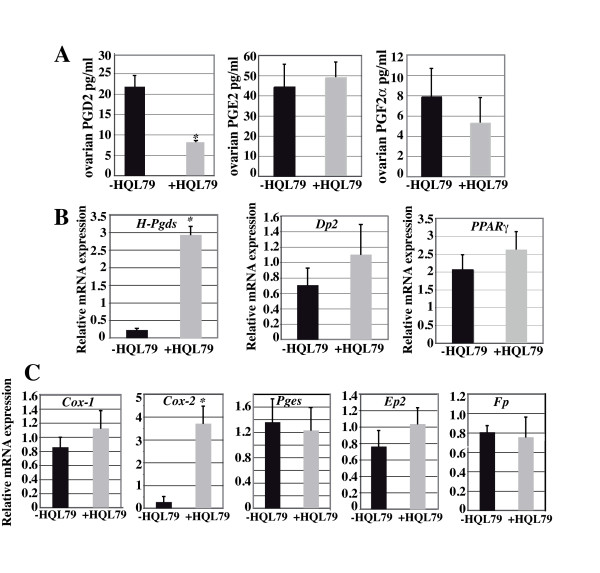
**Prostaglandins synthesis in the ovary upon PGD2 depletion**. (**A**), Levels of PGD2, PGE2 and PGF2α were measured using ELISA in HQL-79 treated or not ovaries (n = 8 for each condition). Results expressed in pg of prostaglandin/ml showed that PGD2 content is significantly decreased (P-value < 0.01) by 65% upon HQL-79 treatment whereas PGE2 and PGF2α contents were not affected; error bars indicate SD of assays done with two dilutions of the eight samples of each group. Expression levels of H-*Pgds, Dp2, PPARγ *(**B**) and *Cox-1, Cox-2, mPges-2, Ep2, Fp *(**C**) in ovaries of HQL-79 treated (n = 8) or not (n = 8) mice. By real time RT-PCR, no significant difference of *Dp2, PPARγ *(**B**) and *Cox-1, mPges-2, Ep2, Fp *(**C**) expression level was detectable whereas a large increase of *Cox-2 *and H-*Pgds *expression level was measured upon HQL-79 treatment. All the expression level values were normalized to those of *Hprt*. Data are expressed as means +/- SE (columns and bars); * P < 0.05 *vs *control.

We evaluated the implication of H-Pgds mediated-PGD2 signaling within the ovarian physiology using the H-Pgds specific inhibitor HQL-79 [36-38]. To confirm the significance of the inhibition by HQL-79 and evaluate the incidence of PGD2 depletion on prostaglandin production, we measured the level of PGD2, PGE2 and PGF2α in ovaries of HQL-79-treated mice. As expected, the ovarian level of PGD2 was markely reduced by 65% in the HQL-79 treated mice compared to that in the untreated mice. However, no significant different levels of PGE2 and PGF2α were measured (Figure 3A).

We then analyzed the PGD2 pathway components and showed by real time RT-PCR that H-*Pgds *expression was up-regulated concomitantly to the reduced level of PGD2 in HQL-79 treated ovaries (Figure 3B). On the other hand, no significantly different expression of the *Dp2 *and *PPAR*γ genes (Figure 3B) was detected upon HQL-79 treatment and no expression of L-*Pgds *and *Dp1 *receptor genes was detected in the control or treated ovaries (data not shown).

To evaluate the impact of the PGD2 signaling on other prostaglandin pathways and considering the importance of PGE2 and PGF2α for ovarian function, we then determined the mRNA contents of cyclooxygenases *Cox1 *and *Cox2*, prostaglandin synthase (membrane-bound) m-*Pges-2*, and the receptors *Ep2 *and *Fp *by quantitative RT-PCR in ovaries from mice (in estrous phase) treated with vehicle or HQL-79. The ovarian *Cox1*, *Pges *and *Ep2, Fp *mRNA levels were not significantly different in the untreated or HQL-79 treated mice (Figure 3C) that were in agreement with the stable levels of PGE2 and PGF2α. However, the expression of *Cox-2 *was significantly increased by 10 fold in HQL-79 treated ovaries compared to control ovaries (Figure 3C).

Taken together, these results indicate that 65% of H-Pgds activity were inhibited by HQL-79 but this treatment has no effect on PGE2 and PGF2α prostaglandin pathways in the ovary; however, the reduced level of PGD2 induces *Cox-2 *gene expression that could contribute to the up-regulation of H-*Pgds *gene expression in order to restore the intraovarian PGD2 content.

### PGD2 signaling is necessary for FSH signaling and steroidogenesis in the mouse ovary

**Figure 4:**
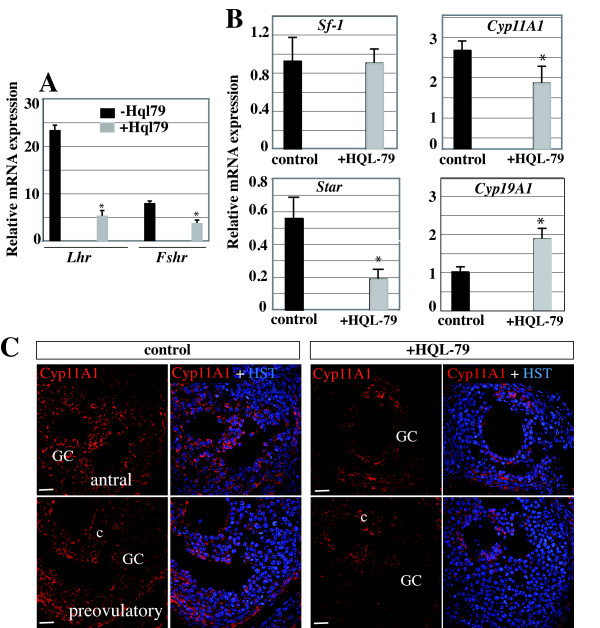
**PGD2 signaling regulates gonadotropin receptors and steroidogenic genes expression**. *FshR *and *LhR *(**A**) and *Sf-1*, *Cyp11A1*, *StAR*, *Cyp19A1 *(**B**) mRNA expression levels were assessed using real time RT-PCR in ovaries from adult cycling mice treated (n = 10) or not (n = 10) using H-Pgds inhibitor HQL-79 (1 mg/kg/day). The values of at least two repeats of two different RT reactions were averaged and normalized to *Gapdh *expression. Values represent mean +/- SEM and * represents significant differences P < 0.025 compared with untreated ovaries (control). (**C**), CYP11A1 protein expression was detected in untreated (control) or treated (+HQL-79) ovaries (in red). Upon HQL-79 treatment, a largely decreased expression is detected in antral and preovulatory follicles. Nuclei are labeled in blue (Hoescht dye, HST). GC: granulosa cells, c: cumulus cells. Scale bars = 50 μm.

Folliculogenesis and synthesis of steroid hormones in the ovary depends on the coordinated actions of FSH and LH acting through their respective receptors FshR and LhR [2]. We thus evaluated the implication of H-Pgds mediated-PGD2 signaling within the gonadotropin pathways. Adult female mice were treated with the H-Pgds inhibitor HQL-79 (at doses 0.1-1 or 10 mg/kg/day) [36-38] or with vehicle for five to nine days until mice reached the estrous phase and the resulting ovaries were examined in terms of their expression of gonadotropin receptors and ovarian markers. For the three doses of HQL-79, the reduced level of H-Pgds produced PGD2 clearly impaired ovarian gonadotropin receptor expression, as shown by the reduction in *FshR *and *LhR *levels by 50% and 80% respectively (data not shown for 0.1 and 10 mg/kg/day and Figure 4A, dose 1 mg/kg/day). Induced steroidogenesis is regulated by increased StAR (steroidogenic acute regulatory) protein expression under the positive control of gonadotropin signaling. StAR is the primary regulator of cholesterol transport into the mitochondria where the steroid precursor is then converted by CYP11A1 side-chain cleavage enzyme (P450scc) to pregnenolone. We demonstrated here that, when compared to levels in the untreated ovary, inhibition of H-Pgds enzymatic activity significantly reduced expression of *StAR *and *Cyp11A1 *genes by 60% and 50% respectively (Figure 4B), whereas PGD2 signaling did not affect expression levels of SF-1, a major activator of steroidogenesis gene expression. In contrast, expression levels of the *Cyp19A1 *gene increased significantly by 30% (Figure 4B). CYP11A1 protein expression was also largely reduced in granulosa cells of the growing follicles of ovaries treated by HQL-79, when compared to that observed in WT ovaries (Figure 4C).

**Figure 5:**
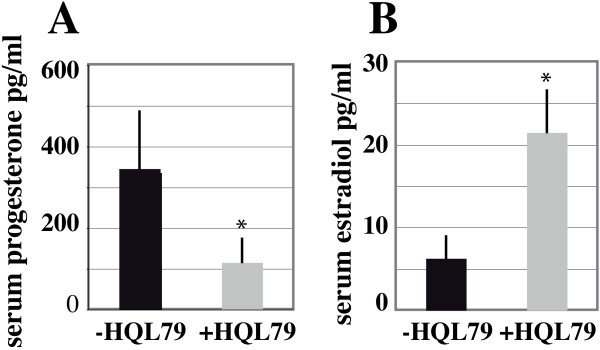
**Progesterone and estradiol production is modified upon H-Pgds enzymatic inhibition**. (**A**), serum progesterone levels. (**B**), serum estradiol levels were measured by Elisa on extracted sera. Bars represent the average of twenty animals (n = 20 for untreated mice and n = 20 for HQL-79 treated mice). HQL-79 treatment induces a 50% decrease of progesterone production and a 50% increase of estradiol production. * represents significant differences P < 0.05, compared to untreated ovaries (-HQL-79).

We next evaluated serum levels of the ovarian steroid hormones estradiol and progesterone in twenty WT and twenty female mice treated with HQL-79 for five to nine days, all in the estrous period. The results showed a significant reduction of 50% in the basal level of progesterone in the mice treated with HQL-79, when compared to that measured in the WT (Figure 5A). In contrast, the estradiol level increased by 50% in the HQL-79 treated mice compared to WT (Figure 5B), following the increased aromatase *Cyp19A1 *expression described above (Figure 4B).

**Figure 6:**
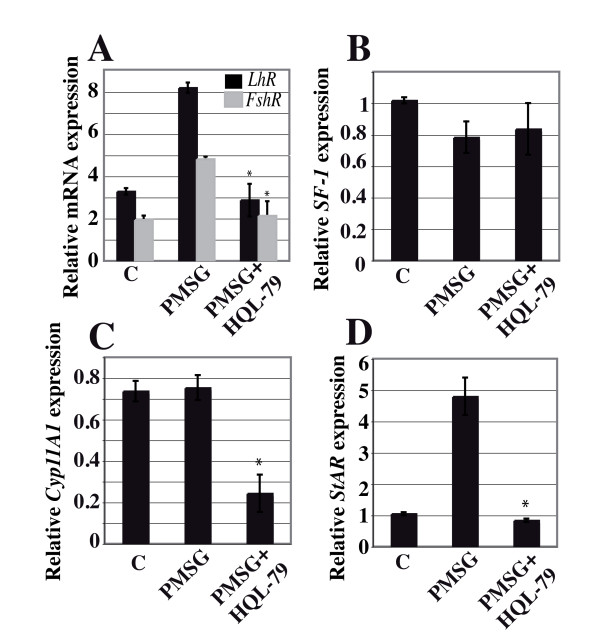
**PGD2 signaling is necessary for FSH action**. Adult cycling female mice were treated with 5 I.U. PMSG without (PMSG) or with (PMSG+HQL-79) administration of HQL-79 inhibitor. *FshR*, *LhR *(**A**), *Sf-1 *(**B**), *Cyp11A1 *(**C**) and *StAR *(**D**) gene expression levels in ovaries (n = 5 for each condition), were analyzed by real-time RT-PCR. The values of at least two repeats of two different RT reactions were averaged and normalized to *Gapdh *expression. Values represent mean +/- SE and * represents significant differences P < 0.05 (**A**), P < 0.001 (**C-D**) compared with ovaries treated with PMSG only.

To evaluate the relationships between PGD2 signaling and FSH action, we stimulated mice with PMSG which mimics the function of FSH. As expected, *FshR *and *LhR *expression was increased by 2.5 fold in PMSG-treated versus untreated control ovaries (Figure 6A). Accordingly, this stimulation was inhibited upon co-treatment with the HQL-79 inhibitor (Figure 6A), indicating the requirement for intact PGD2 signaling in order for PSMG to take effect. Subsequently, inhibition of H-Pgds activity also inhibited *StAR *expression induced after PMSG treatment (Figure 6D) whereas *Cyp11A1 *expression decreased after HQL-79 treatment (Figure 6C), confirming that PGD2 is involved in *Cyp11A1 *activation. On the other hand, *SF-1 *expression level remained independent of PMSG and HQL-79 treatment (Figure 6B).

### H-Pgds-produced PGD2 is implicated in the control of granulosa cell proliferation

**Figure 7:**
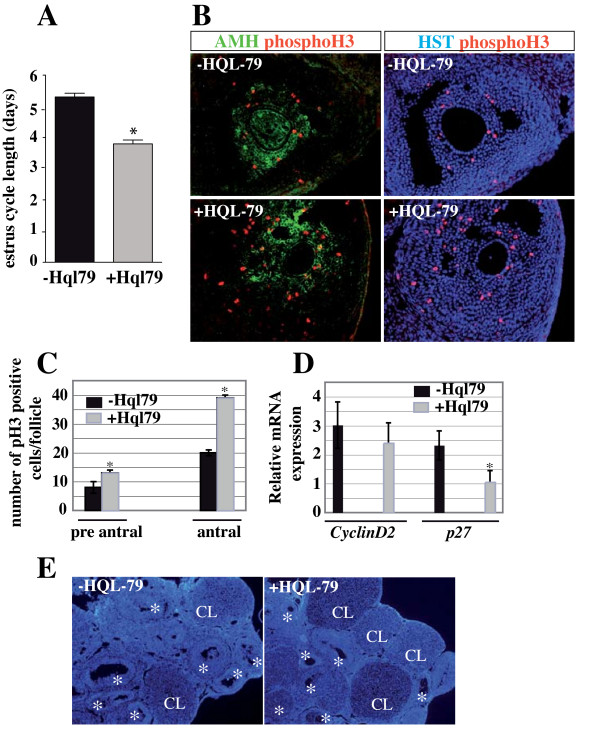
**PGD2 signaling controls the granulosa cell proliferation**. (**A**), The length of estrous cycles in five WT and five HQL-79-treated adult mice were assessed in vaginal smears collected every day for 16 consecutive days. Results of the five animals were averaged and were expressed as means +/- SE (colums and bars), * P value = 0.0097. (**B**), Proliferation of granulosa cells of antral follicles was assessed using immunofluorescence with mitosis marker phosphohistone H3 (phosphoH3) antibody (in red) on cryosections of wild type (-HQL-79) or HQL-79 (+HQL-79) treated ovaries; granulosa cells were identified by anti-Müllerian hormone (AMH) antibody (in green) and nuclei were labeled by the Hoescht Dye (HST) (in blue). Numbers of phospho-H3-positive cells were determined on ten independent fields of three different ovaries for each condition and are represented on the graphs (**C**). * represents significant increased number of mitotic cells in HQL-79 treated compared to that in untreated ovaries. (**D**), *CyclinD2 *and *p27 *expression levels in five wild type and five HQL-79-treated ovaries were quantified by real time RT-PCR and were normalized to *Gapdh *expression. Values are the result of averaged experiments (done in triplicate) on the five independent ovaries. * represents the significant decrease of p27 expression in HQL-79 compared to that in untreated ovaries (P-value < 0.025). **(E)**, The follicular content of HQL-79 treated ovaries (at their proestrous stage) were compared to that of WT ovaries by labeling sections with the Hoescht dye. CL: corpora lutea, * growing follicles.

We assessed the length of estrous cycles in five WT and five HQL-79-treated adult mice using vaginal smears collected over 16 consecutive days (three to four cycles). The WT mice (-HQL-79) had cyclical estrous cycles lasting more than five days (5.3 days) whereas in contrast, HQL-79 treated (+HQL-79) mice had significantly shorter cycles lasting less than four days (3.8 days) (Figure 7A, P-value: 0.0097). To characterize the observed changes of inactivation of H-Pgds activity at the cellular level, we examined the proliferation rate of granulosa cells (GCs) in the developing follicles. GCs partially depleted of PGD2 signaling showed an increased proliferation upon immunostaining for mitosis marker phosphohistone H3 (phosphoH3) (Figure 7B). A significant increase of 30% in granulosa cell proliferation was seen in the pre-antral follicles and reached 50% in the GCs of antral follicles of HQL-79 treated ovaries, compared to untreated ovaries (Figure 7C). In contrast, apoptosis in the GCs of the growing follicles was not modified by the lack of PGD2 signaling (data not shown). As shown in Figure 7D, this increase in cell proliferation is associated with a significantly decreased expression of *CDKN1B *(p27) in the treated ovaries, whereas levels of *CyclinD2 *expression remained unmodified. Consequently, the number of corpora lutea in HQL-79 ovaries was increased by two fold compared to that in untreated ovaries (Figure 7E) (female mice at the proestrous phase of their cycle), suggesting that upon HQL-79 treatment, the number of growing and maturating follicles have increased. Collectively, these results support the hypothesis where PGD2 signaling negatively impacts GC proliferation *in vivo*, thus promoting conditions favoring granulosa cell differentiation and subsequently steroidogenesis.

## Discussion

In this study, we describe the expression of *H-Pgds *mRNA in the adult mouse ovary. This localization includes granulosa cells from growing follicles through primary to antral and pre-ovulatory stages, and the corpus luteum formed after ovulation. H-Pgds is thus the sole source of PGD2 in the ovary since the second enzyme able to produce PGD2 (L-Pgds) is not expressed [19]. In the embryonic gonad, L-Pgds secreted PGD2 signals through the adenylcyclase-coupled receptor DP1 to activate expression of the Sertoli cell differentiating gene *Sox9 *and contribute to the nuclear translocation of SOX9 protein [19,30]. In the adult ovary, the Ca^++ ^coupled DP2 receptor is exclusively expressed in granulosa cells. Considering how Sertoli and granulosa cells have common ancestor precursor cells [42], this differential expression of both receptors and the dual functional convergence between L- and H-Pgds might constitute part of the antagonistic regulation between male and female pathways [43,44] and be a key regulatory step in maintaining the differentiation of both Sertoli and granulosa cell types [45]. PGD2 is metabolized to 15d-PGJ2, the high affinity natural ligand for the PPARγ receptor expressed in granulosa cells of developing follicles [46,47]. These results thus suggest that both receptors DP2 and PPARγ might relay PGD2 signaling in the adult ovary.

The process of granulosa cell differentiation occurring throughout progression from a pre-antral to pre-ovulatory follicle is dependent on sufficient FSH stimulation [48,49] and is marked by the acquisition of *FshR *and *LhR *expression and increased steroidogenesis. In this study, we demonstrated that H-Pgds enzymatic activity is required in order for FSH to regulate expression of both *FshR *and *LhR *receptors, suggesting PGD2 to be an autocrine positive regulator of *FshR *and *LhR *expression in the ovary. This regulation may act directly on the FSH-induced *FshR *promoter activity as in the case of inhibin-A [50], or might otherwise act indirectly by increasing *FshR *mRNA stability, as in the case of IGF-I [51]. The inhibition of H-Pgds enzymatic activity leads to a decrease in *FshR *and *LhR *expression but does not affect that of *SF-1*, the major activator of steroidogenesis gene expression [52]. This supports the implication of PGD2 signaling in the FSH-induced expression of the *StAR *gene, independently on SF-1. SF-1 is essential for the development and function of the reproductive axis at multiple levels [52] and FSH has been shown to activate SF-1-mediated transcription using various mechanisms [53]. Thus, regulation of *FshR *expression might be one of the causes of *LhR *and steroidogenic gene down-regulation, and of the decrease in progesterone production upon PGD2 signaling inhibition [54].

In contrast, following the decrease in *Cyp11A1 *and *StAR *expression levels upon PGD2 depletion, we found that levels of both aromatase expression and serum estradiol increased in treated female mice compared to untreated animals. On the other hand, we observed that granulosa cells partially depleted of PGD2 signaling show increased proliferation based on immunostaining for mitosis marker phosphohistone H3, which we confirmed at the molecular level through the significantly decreased expression of *CDKN1B *(p27). This increased proliferation lead to an increased number of the maturating follicles that might explain the higher levels of *Cyp19A1 *mRNA expression and secreted estradiol upon HQL-79 treatment, rather than being a consequence of the *Cox-2 *up-regulation that was detected in HQL-79 ovaries. The up-regulation of *Cyp19A1 *gene expression via COX-2 was shown to be dependent on PGE2 synthesis and cAMP signaling in undifferentiated rat granulosa cells [9] or in human breast tumor cells [55]. Our data showed that *Cox-2 *expression is up-regulated, however, PGE2 synthesis was not modified. Indeed, the side-effect of HQL-79 treatment (i.e. increased PGE2 production) [26] related in the lung tissues of sensitized guinea pigs [56] was not detected in our system as it has not been seen in sheep vesicular gland microsomes [56] or *in vivo *in H-*Pgds *transgenic mouse strain [36].

In this study, we measured high levels of *Cox-2 *and H-*Pgds *transcripts whereas no modification of *Cox-1 *has been measured in HQL-79 treated ovaries. The functional coupling between H-Pgds/Cox-1 or H-Pgds/Cox-2 has been demonstrated respectively, in the immediate or the delayed response in mast cells during the cytokine stimulation [38], even though tightly coupling between H-Pgds and Cox-1 is preferentially documented [36,57]. The up-regulation of Cox-2 associated with the down-regulation of H-Pgds protein expression upon HQL-79 treatment has been previously described in the mouse ischemic brain [58]. In the ovary, we can assume that partial depletion of PGD2 might induce *Cox-2 *gene expression that in turn, might activate H-Pgds expression in order to restore the intraovarian PGD2 content. PGJ2, a PGD2 metabolite was shown to inhibit osteoblastic differentiation through PPARγ activation and down-regulation of Cox-2 [59]. This process would take place without any interaction with other prostanoid-synthetizing mechanisms as it has been previously reported in other systems, induction of fever [60] or induction of inflammation in muscle necrosis [61], since PGE2 and PGF2α prostaglandin pathways are not modified upon HQL-79 treatment.

Using the H-Pgds specific inhibitor HQL-79 known to exactly mimic the phenotype of *H-Pgds *KO mice in various systems such as inflammation, muscle necrosis [31,38], we identify an important and unappreciated role for PGD2 signaling in modulating the balance of proliferation, differentiation and steroidogenic activity of the granulosa cells, through both FSH dependent and independent mechanisms. Thus, these results suggest PGD2 as a modulator of follicle development, even though no reproductive defects have been reported in female H-*Pgds *KO mice [31,62]. The physiological importance of PGD2 for ovarian function and normal female fertility might be assessed in this mouse strain or in mice conditionnally invalidated for H-Pgds in the ovary under the control of Anti-Müllerian hormone (Amh) promoter (Amh-cre, [63]) to overcome a putative central effect of H-Pgds produced PGD2.

## Abbreviations

FSH: follicle-stimulating hormone; LH: luteinizing hormone; Cox-2: cyclooxygenase-2; CYP11A1: cytochrome P450 11A1 (P450scc: Cholesterol-side chain cleavage enzyme); CYP19A1: cytochrome P450 19A1 (aromatase); StAR: steroidogenic acute regulatory protein; SF-1: steroidogenic factor 1; PGD2: prostaglandin D2; PGE2: prostaglandin E2; PGF2α: prostaglandin F2α; PPARγ: peroxisome proliferator-activated receptor gamma; PMSG: pregnant mare serum gonadotropin.

## Competing interests

The authors declare that they have no competing interests.

## Authors' contributions

All authors read and approved the final manuscript. Conceived and designed the experiments: FP, BBB. Performed the experiments: AF, PP, FP, BBB. Analyzed the data: AF, PP, CS, FP, BBB. Contributed reagents/materials/analysis tools: CS, FP, BBB. Wrote the paper: BBB.

## References

[B1] Richards JS Fitzpatrick SL Clemens JW Morris JK Alliston T Ovarian cell differentiation: a cascade of multiple hormones, cellular signals, and regulated genes Recent Prog Horm Res 1995 50 223 254 7740159 10.1016/b978-0-12-571150-0.50014-7

[B2] Fortune JE The early stages of follicular development: activation of primordial follicles and growth of preantral follicles Anim Reprod Sci 2003 78 135 163 10.1016/S0378-4320(03)00088-5 12818642

[B3] Challis JR Prostaglandins and reproduction--what do knockouts really tell us? Nat Med 1997 3 1326 1327 10.1038/nm1297-1326 9396601

[B4] Cha YI Solnica-Krezel L DuBois RN Fishing for prostanoids: deciphering the developmental functions of cyclooxygenase-derived prostaglandins Dev Biol 2006 289 263 272 10.1016/j.ydbio.2005.10.013 16310177

[B5] Lim H Paria BC Das SK Dinchuk JE Langenbach R Multiple female reproductive failures in cyclooxygenase 2-deficient mice Cell 1997 91 197 208 10.1016/S0092-8674(00)80402-X 9346237

[B6] Kennedy CR Zhang Y Brandon S Guan Y Coffee K Salt-sensitive hypertension and reduced fertility in mice lacking the prostaglandin EP2 receptor Nat Med 1999 5 217 220 10.1038/7426 9930871

[B7] Sirois J Richards JS Purification and characterization of a novel, distinct isoform of prostaglandin endoperoxide synthase induced by human chorionic gonadotropin in granulosa cells of rat preovulatory follicles J Biol Chem 1992 267 6382 6388 1556140

[B8] Hizaki H Segi E Sugimoto Y Hirose M Saji T Abortive expansion of the cumulus and impaired fertility in mice lacking the prostaglandin E receptor subtype EP(2) Proc Natl Acad Sci USA 1999 96 10501 10506 10.1073/pnas.96.18.10501 10468638 PMC17918

[B9] Cai Z Kwintkiewicz J Young ME Stocco C Prostaglandin E2 increases cyp19 expression in rat granulosa cells: implication of GATA-4 Mol Cell Endocrinol 2007 263 181 189 10.1016/j.mce.2006.09.012 17097802 PMC1779458

[B10] Arosh JA Banu SK Chapdelaine P Madore E Sirois J Prostaglandin biosynthesis, transport, and signaling in corpus luteum: a basis for autoregulation of luteal function Endocrinology 2004 145 2551 2560 10.1210/en.2003-1607 14736737

[B11] Challis JR Calder AA Dilley S Forster CS Hillier K Production of prostaglandins E and Falpha by corpora lutea, corpora albicantes and stroma from the human ovary J Endocrinol 1976 68 401 408 10.1677/joe.0.0680401 815508

[B12] Sugimoto Y Yamasaki A Segi E Tsuboi K Aze Y Failure of parturition in mice lacking the prostaglandin F receptor Science 1997 277 681 683 10.1126/science.277.5326.681 9235889

[B13] Challis JR Lye SJ Gibb W Prostaglandins and parturition Ann N Y Acad Sci 1997 828 254 267 10.1111/j.1749-6632.1997.tb48546.x 9329846

[B14] Challis JR Sloboda DM Alfaidy N Lye SJ Gibb W Prostaglandins and mechanisms of preterm birth Reproduction 2002 124 1 17 10.1530/rep.0.1240001 12090913

[B15] Weems CW Weems YS Randel RD Prostaglandins and reproduction in female farm animals Vet J 2006 171 206 228 10.1016/j.tvjl.2004.11.014 16490704

[B16] Fortune JE Willis EL Bridges PJ Yang CS The periovulatory period in cattle: progesterone, prostaglandins, oxytocin and ADAMTS proteases Anim Reprod 2009 6 60 71 20390049 PMC2853051

[B17] Breyer RM Bagdassarian CK Myers SA Breyer MD Prostanoid receptors: subtypes and signaling Annu Rev Pharmacol Toxicol 2001 41 661 690 10.1146/annurev.pharmtox.41.1.661 11264472

[B18] Matsuoka T Hirata M Tanaka H Takahashi Y Murata T Prostaglandin D2 as a mediator of allergic asthma Science 2000 287 2013 2017 10.1126/science.287.5460.2013 10720327

[B19] Malki S Nef S Notarnicola C Thevenet L Gasca S Prostaglandin D2 induces nuclear import of the sex-determining factor SOX9 via its cAMP-PKA phosphorylation Embo 2005 J 24 1798 1809 10.1038/sj.emboj.7600660 PMC1142593 15889150

[B20] Moniot B Declosmenil F Barrionuevo F Scherer G Aritake K The PGD2 pathway, independently of FGF9, amplifies SOX9 activity in Sertoli cells during male sexual differentiation Development 2009 136 1813 1821 10.1242/dev.032631 19429785 PMC4075598

[B21] Boie Y Sawyer N Slipetz DM Metters KM Abramovitz M Molecular cloning and characterization of the human prostanoid DP receptor J Biol Chem 1995 270 18910 18916 10.1074/jbc.270.32.18910 7642548

[B22] Breyer MD Breyer RM G protein-coupled prostanoid receptors and the kidney Annu Rev Physiol 2001 63 579 605 10.1146/annurev.physiol.63.1.579 11181968

[B23] Toth B Hornung D Scholz C Djalali S Friese K Peroxisome proliferator-activated receptors: new players in the field of reproduction Am J Reprod Immunol 2007 58 289 310 10.1111/j.1600-0897.2007.00514.x 17681045

[B24] Urade Y Eguchi N Lipocalin-type and hematopoietic prostaglandin D synthases as a novel example of functional convergence Prostaglandins Other Lipid Mediat 2002 68-69 375 382 10.1016/S0090-6980(02)00042-4 12432930

[B25] Urade Y Hayaishi O Biochemical, structural, genetic, physiological, and pathophysiological features of lipocalin-type prostaglandin D synthase Biochim Biophys Acta 2000 1482 259 271 10.1016/S0167-4838(00)00161-8 11058767

[B26] Kanaoka Y Urade Y Hematopoietic prostaglandin D synthase Prostaglandins Leukot Essent Fatty Acids 2003 69 163 167 10.1016/S0952-3278(03)00077-2 12895599

[B27] Shimizu T Yamashita A Hayaishi O Specific binding of prostaglandin D2 to rat brain synaptic membrane. Occurrence, properties, and distribution J Biol Chem 1982 257 13570 13575 6292197

[B28] Nishida Y Yoshioka M St-Amand J The top 10 most abundant transcripts are sufficient to characterize the organs functional specificity: evidences from the cortex, hypothalamus and pituitary gland Gene 2005 344 133 141 10.1016/j.gene.2004.09.007 15656980

[B29] Adams IR McLaren A Sexually dimorphic development of mouse primordial germ cells: switching from oogenesis to spermatogenesis Development 2002 129 1155 1164 11874911 10.1242/dev.129.5.1155

[B30] Wilhelm D Palmer S Koopman P Sex determination and gonadal development in mammals Physiol Rev 2007 87 1 28 10.1152/physrev.00009.2006 17237341

[B31] Mohri I Taniike M Taniguchi H Kanekiyo T Aritake K Prostaglandin D2-mediated microglia/astrocyte interaction enhances astrogliosis and demyelination in twitcher J Neurosci 2006 26 4383 4393 10.1523/JNEUROSCI.4531-05.2006 16624958 PMC6673986

[B32] Michimata T Tsuda H Sakai M Fujimura M Nagata K Accumulation of CRTH2-positive T-helper 2 and T-cytotoxic 2 cells at implantation sites of human decidua in a prostaglandin D(2)-mediated manner Mol Hum Reprod 2002 8 181 187 10.1093/molehr/8.2.181 11818521

[B33] Shiue YL Chen LR Chen CF Chen YL Ju JP Identification of transcripts related to high egg production in the chicken hypothalamus and pituitary gland Theriogenology 2006 66 1274 1283 10.1016/j.theriogenology.2006.03.037 16725186

[B34] Chen LR Lee SC Lin YP Hsieh YL Chen YL Prostaglandin-D synthetase induces transcription of the LH beta subunit in the primary culture of chicken anterior pituitary cells via the PPAR signaling pathway Theriogenology 2010 73 367 382 10.1016/j.theriogenology.2009.09.020 19954828

[B35] Bennegard B Hahlin M Hamberger L Luteotropic effects of prostaglandins I2 and D2 on isolated human corpora luteum Fertil Steril 1990 54 459 464 2168845

[B36] Aritake K Kado Y Inoue T Miyano M Urade Y Structural and functional characterization of HQL-79, an orally selective inhibitor of human hematopoietic prostaglandin D synthase J Biol Chem 2006 281 15277 15286 10.1074/jbc.M506431200 16547010

[B37] Matsushita N Hizue M Aritake K Hayashi K Takada A Pharmacological studies on the novel antiallergic drug HQL-79: I. Antiallergic and antiasthmatic effects in various experimental models Jpn J Pharmacol 1998 78 1 10 10.1254/jjp.78.1 9804056

[B38] Satoh T Moroi R Aritake K Urade Y Kanai Y Prostaglandin D2 plays an essential role in chronic allergic inflammation of the skin via CRTH2 receptor J Immunol 2006 177 2621 2629 16888024 10.4049/jimmunol.177.4.2621

[B39] Malki S Berta P Poulat F Boizet-Bonhoure B Cytoplasmic retention of the sex-determining factor SOX9 via the microtubule network Exp Cell Res 2005 309 468 475 10.1016/j.yexcr.2005.07.005 16087173

[B40] Cherradi N Chambaz EM Defaye G Organization of 3 beta-hydroxysteroid dehydrogenase/isomerase and cytochrome P450scc into a catalytically active molecular complex in bovine adrenocortical mitochondria J Steroid Biochem Mol Biol 1995 55 507 514 10.1016/0960-0760(95)00199-9 8547175

[B41] Moniot B Boizet-Bonhoure B Poulat F Male specific expression of lipocalin-type prostaglandin D synthase (cPTGDS) during chicken gonadal differentiation: relationship with cSOX9 Sex Dev 2008 2 96 103 10.1159/000129694 18577876

[B42] Albrecht KH Eicher EM Evidence that Sry is expressed in pre-Sertoli cells and Sertoli and granulosa cells have a common precursor Dev Biol 2001 240 92 107 10.1006/dbio.2001.0438 11784049

[B43] Schlessinger D Garcia-Ortiz JE Forabosco A Uda M Crisponi L Determination and Stability of Gonadal Sex J Androl 2009 19875493 10.2164/jandrol.109.008201 PMC2882171

[B44] Wilhelm D Washburn LL Truong V Fellous M Eicher EM Antagonism of the testis- and ovary-determining pathways during ovotestis development in mice Mech Dev 2009 126 324 336 10.1016/j.mod.2009.02.006 19269320 PMC2680453

[B45] Piprek RP Molecular mechanisms underlying female sex determination--antagonism between female and male pathway Folia Biol (Krakow) 2009 57 105 113 10.3409/fb57_3-4.105-113 19777952

[B46] Komar CM Braissant O Wahli W Curry TE Jr Expression and localization of PPARs in the rat ovary during follicular development and the periovulatory period Endocrinology 2001 142 4831 4838 10.1210/en.142.11.4831 11606451

[B47] Komar CM Peroxisome proliferator-activated receptors (PPARs) and ovarian function--implications for regulating steroidogenesis, differentiation, and tissue remodeling Reprod Biol Endocrinol 2005 3 41 10.1186/1477-7827-3-41 16131403 PMC1266036

[B48] Kumar TR Wang Y Lu N Matzuk MM Follicle stimulating hormone is required for ovarian follicle maturation but not male fertility Nat Genet 1997 15 201 204 10.1038/ng0297-201 9020850

[B49] Richards JS Pangas SA The ovary: basic biology and clinical implications J Clin Invest 2010 120 963 972 10.1172/JCI41350 20364094 PMC2846061

[B50] Lu C Yang W Chen M Liu T Yang J Inhibin A inhibits follicle-stimulating hormone (FSH) action by suppressing its receptor expression in cultured rat granulosa cells Mol Cell Endocrinol 2009 298 48 56 10.1016/j.mce.2008.09.039 18992787

[B51] Minegishi T Hirakawa T Kishi H Abe K Abe Y A role of insulin-like growth factor I for follicle-stimulating hormone receptor expression in rat granulosa cells Biol Reprod 2000 62 325 333 10.1095/biolreprod62.2.325 10642569

[B52] Bakke M Zhao L Hanley NA Parker KL SF-1: a critical mediator of steroidogenesis Mol Cell Endocrinol 2001 171 5 7 10.1016/S0303-7207(00)00384-1 11165004

[B53] Jeyasuria P Ikeda Y Jamin SP Zhao L De Rooij DG Cell-specific knockout of steroidogenic factor 1 reveals its essential roles in gonadal function Mol Endocrinol 2004 18 1610 1619 10.1210/me.2003-0404 15118069

[B54] Yazawa T Inanoka Y Okada R Mizutani T Yamazaki Y PPAR-{gamma} Coactivator-1{alpha} Regulates Progesterone Production in Ovarian Granulosa Cells with SF-1 and LRH-1 Mol Endocrinol10.1210/me.2009-0352 PMC5419099 20133449

[B55] Prosperi JR Robertson FM Cyclooxygenase-2 directly regulates gene expression of P450 Cyp19 aromatase promoter regions pII, pI.3 and pI.7 and estradiol production in human breast tumor cells Prostaglandins Other Lipid Mediat 2006 81 55 70 10.1016/j.prostaglandins.2006.07.003 16997132

[B56] Matsushita N Aritake K Takada A Hizue M Hayashi K Pharmacological studies on the novel antiallergic drug HQL-79: II. Elucidation of mechanisms for antiallergic and antiasthmatic effects Jpn J Pharmacol 1998 78 11 22 10.1254/jjp.78.11 9804057

[B57] Ueno N Takegoshi Y Kamei D Kudo I Murakami M Coupling between cyclooxygenases and terminal prostanoid synthases Biochem Biophys Res Commun 2005 338 70 76 10.1016/j.bbrc.2005.08.152 16140261

[B58] Liu M Eguchi N Yamasaki Y Urade Y Hattori N Protective role of hematopoietic prostaglandin D synthase in transient focal cerebral ischemia in mice Neuroscience 2009 163 296 307 10.1016/j.neuroscience.2009.06.027 19531375

[B59] Liu M Eguchi N Yamasaki Y Urade Y Hattori N Focal cerebral ischemia/reperfusion injury in mice induces hematopoietic prostaglandin D synthase in microglia and macrophages Neuroscience 2007 145 520 529 10.1016/j.neuroscience.2006.12.018 17241746

[B60] Gao W Schmidtko A Lu R Brenneis C Angioni C Prostaglandin D(2) sustains the pyrogenic effect of prostaglandin E(2) Eur J Pharmacol 2009 608 28 31 10.1016/j.ejphar.2009.01.051 19249295

[B61] Mohri I Aritake K Taniguchi H Sato Y Kamauchi S Inhibition of prostaglandin D synthase suppresses muscular necrosis Am J Pathol 2009 174 1735 1744 10.2353/ajpath.2009.080709 19359520 PMC2671262

[B62] Trivedi SG Newson J Rajakariar R Jacques TS Hannon R Essential role for hematopoietic prostaglandin D2 synthase in the control of delayed type hypersensitivity Proc Natl Acad Sci USA 2006 103 5179 5184 10.1073/pnas.0507175103 16547141 PMC1458814

[B63] Lecureuil C Fontaine I Crepieux P Guillou F Sertoli and granulosa cell-specific Cre recombinase activity in transgenic mice Genesis 2002 33 114 118 10.1002/gene.10100 12124943

